# A case of completed course multifocal osteonecrosis (MFON) during pregnancy due to primary antiphospholipid syndrome

**DOI:** 10.1186/s43166-022-00122-4

**Published:** 2022-03-24

**Authors:** Aya El Shintenawy, Soha Khallaf, Esraa El Shentenawy, Ali El Deeb

**Affiliations:** Department of Rheumatology, Rehabilitation & Physical medicine Tanta University, Tanta, Egypt

**Keywords:** Osteonecrosis, Primary antiphospholipid syndrome, Hips, Shoulders

## Abstract

Osteonecrosis of both shoulders and hips is a rare presentation of primary antiphospholipid syndrome. A female patient aged 23 years old with no systemic diseases has her only complaint which was pain and limitations in both hips followed by both shoulders. Careful detailed history and clinical examination is essential for reaching optimum diagnosis thus good management. MRI for hip and shoulder joints is essential for the diagnosis of osteonecrosis. Exclusion of all causes of secondary osteonecrosis by history, clinical examination, and laboratory studies should be done before diagnosing the rare causes of osteonecrosis or the primary type. Pregnancy is an exacerbation factor for primary osteonecrosis. We recommend that the female patient with primary osteonecrosis should receive prophylactic antithrombotic during pregnancy. In conclusion, exclusion of all secondary causes of osteonecrosis is mandatory to reach an accurate diagnosis.

## Background

Patient only presented with MFON, 23 years old from El Mahalla Gharbia Governorate, presented with pain and limitation of both hips and then shoulders after exclusion of all secondary causes patient was diagnosed as primary APLS.

### Conclusion

Exclusion of all secondary causes of osteonecrosis is mandatory to reach an accurate diagnosis.

## Case presentation

Female patient aged 23 years old from El Mahalla Gharbia Egypt, married with one offspring aged 6 months old with no special habits of medical importance.

- Patient complaint started 3 years ago with gradual onset of pain in her right hip then after 6 months of pain started in her left hip. She sought medical advice and received NSAIDs for relief of pain. One year later, patient started to notice a limitation of movement of her right hip followed by the left hip.

**Fig. 1:**
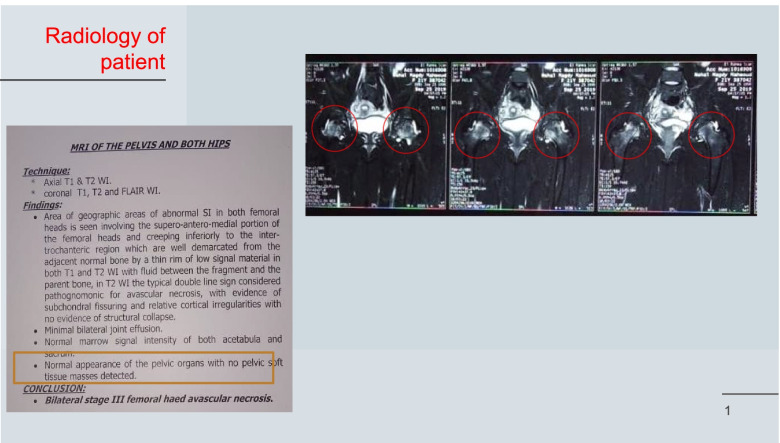
MRI of both hips revealed abnormal signals in the superior-antero-medial portion of both femoral heads and inferiorly to the intertrochanteric region which are well demarcated from the adjacent normal bone by a thin rim of low signal material in both T1 and T2 WI with fluid between the fragment and the bone. T2 WI revealed the typical double line sign which is considered pathognomonic for avascular necrosis with evidence of subchondral fissuring and relative cortical irregularities with no evidence of structural collapse. *Conclusion*: bilateral stage III femoral head avascular necrosis

There was no improvement of pain with NSAIDs, so the patient sought medical advice (orthopedic consultation) and was requested plain X-rays then MRI for both hip joints and his diagnosis was avascular necrosis of both hip joints (Fig. 1).

- Two months later, the patient did an operation in the right hip and then left hip (2 months apart) in the form of core decompression. After the operations, there was an improvement of pain and mild improvement of hip movements.

The patient got pregnant and delivered a healthy full-term newborn 6 months ago. During pregnancy, she started to feel gradual onset of pain in her right shoulder on week 28 then in the left shoulder on week 30; also, the pain of her both hips worsened in the late of her pregnancy by week 32 and postpartum; during pregnancy, the patient prescribed the pain as being so severe that interferes with her daily activity.

**Fig. 2:**
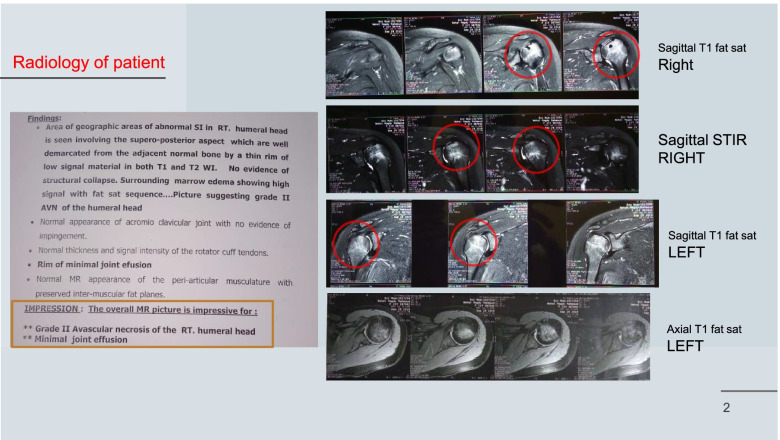
MRI of both shoulders revealed abnormal signals in the right humeral head involving the superior-posterior aspect which are well demarcated from the adjacent normal bony by a thin rim of low signal material in both T1 and T2 WI. No evidence of structural collapse with marrow edema showing high signal with fat sat sequence. *Conclusion:* grade II AVN of the right humeral head and grade I AVN of the left humeral head with minimal joint effusion

- She received NSAIDs for pain relief. The patient again sought medical advice (orthopedic consultation) who in return requested plain X-rays and MRI for both shoulder joints and his diagnosis was avascular necrosis of both shoulder joints (Fig. 2). Then, the patient was referred to the rheumatology and rehabilitation outpatient clinic, Tanta University.
- Pain increases with daily activity and decreases with rest and NSAIDs. There is no history of fever, rash, photosensitivity, and oral or genital ulcers.

Present history:

- No history of trauma
- No history of drug intake especially corticosteroids.
- There is no history of other joint involvement either peripheral or axial.
- No morning stiffness.
- No history of hair fall.
- No ocular complaint.
- No Raynaud’s phenomenon.
- No history of skin lesions.
- Family history:
- Negative consanguinity
- No history of rheumatological diseases in her family
- No history of FMF

Physical examination:

- Patient is well alert, oriented, cooperative, and independent.
- Vital signs are normal.
- Other joints either peripheral or axial are normal.
- No enthesitis.

Both shoulder joints—tenderness and limitation of ROM especially internal rotation.

Both hip joints—tenderness and limitation of ROM especially abduction and internal rotation.

Investigations: Table 1 shows the investigations done.

**Table 1:** Investigations

Investigation	
Hematology CBC shows Hb: 11.9 gm/dl. PLTs: 260 000 /cmm. TLC: 6400/cmm (mild microcytic hypochromic anemia).	
TSH: 1.13 iu/ml Free t3 3.18 pg/ml. Free t4: 1.23 ng/dl.	
Inflammatory markers: ESR 1st hour: 24 mm/h. ESR 2nd hour: 48 mm/h. CRP: 6.5 mg/l.	
Total lipid profile Cholesterol: 151 mg/dl. TG: 44 mg/dl. HDL-C: 65 mg/dl. LDL-C: 77 mg/dl.	
Renal function Serum urea: 30 mg/dl. Serum creatinine: 0.8 mg/dl.	
Liver function tests SGOT: 23 U/L. SGPT: U/L. Viral markers: negative.	
ANA: negative Anti-DsDNA: negative Anti-sm: negative C3&4: Normal not consumed Anti Ro/La : negative -Anticentromere, anti-scleroderma 70: negative. Antiphospholipid antibodies: LA: Positive 55 s (moderate elevated) -B2 Glycoprotein IgG, IgM : 5.5,5.8 u/ml. – aCL: negative. Test was reevaluated after 12 weeks and revealed positive LA and B2 glycoprotein IgG, IgM. - Protein C& S: normal - Parathormone: normal (40 pg/ml) - Serum cortisol level: normal (20mcg/dl) - Antithrombin: normal.	

Causes of multifocal osteonecrosis are presented in Table 2 [1].

**Table 2:** Causes of osteonecrosis [1]

Non-rheumatological causes	Rheumatological causes
1. Corticosteroid use.	1. SLE.
2. Trauma.	2. Sjogren syndrome. 3. Inflammatory bowel disease.
3. Alcohol intake.	
4. Gauchers disease.	4. Systemic sclerosis.
5. Sickle cell anemia.	5. Antiphospholipid syndrome.
6. HIV infection.	
7. Caison disease.	
8. Thrombophilic disorders.	

Final diagnosis: a case of multifocal osteonecrosis due to primary antiphospholipid syndrome.

Treatment of case: the patient was considered high risk so treatment according to 2019 EULAR recommendation [2]: Vitamin K antagonist (warfarin) with INR target 3–4. The patient is now on 5 mg once daily.

## Discussion

Multifocal osteonecrosis is defined by the involvement of 3 or more anatomic sites. It is unusually observed in only 3–11% of patients with osteonecrosis. The most common sites affected are the femoral head, knee, and humeral head respectively [3].

Main rheumatologic diseases associated with MFON are SLE, IBD, secondary APLS, Sjogren syndrome, systemic sclerosis, and Behcet. It is not common in primary antiphospholipid [4].

Hip pain during the later stages of pregnancy and during the postpartum period is a common presentation and usually misdiagnosed as sciatica, pelvic structural compression, and lumbosacral strain [5]. Transient osteoporosis, which is self-limiting and typically resolves within months, and osteonecrosis, which results in femoral head collapse and degenerative changes in the joint, can also cause pain [5].

The etiology of osteonecrosis of the femoral head during pregnancy is still largely unknown. However, theories have been proposed suggesting the pathogenesis is likely to be multifactorial including hormonal, mechanical, and coagulation factors [6]. Venous congestion and hypercoagulability are common in the third trimester during pregnancy. Other possible etiologies are ovarian hyperstimulation drugs, which have the detrimental effects of hyperviscosity and hypercoagulability, and the mechanical stress or overload by excessive labor and weight gain during the last trimester of pregnancy [6]. There are many endocrine modifications that occur during pregnancy as parathyroid hyperplasia and the excess production of estrogen and progesterone by the placenta [7] leading to destabilization of endogenous plasma lipoproteins and lipid metabolism in the liver, which could promote fat embolism. Also, an increase in estrogen and progesterone results in increased adrenocortical activity and levels of adrenal corticosteroids to almost three times the level of a non-pregnant woman [7].

Hormonal/ drug-induced osteonecrosis includes steroid-induced osteonecrosis. Zhang et al. [8] reviewed 43 cases of steroid-induced osteonecrosis following the SARS epidemic and suggested that a single dose of 200 mg of methylprednisone or a cumulative dose of more than 4000 mg was a significant risk factor for the development of multifocal osteonecrosis. Gunal and Karatosun [9] showed bilateral osteonecrosis of the hip after a single dose (75.5 mg) for treatment of an allergic reaction. Mckee et al. [10] reviewed 15 cases of osteonecrosis with a mean of 20.5 days of treatment and doses of up to 3300 mg of prednisone.

There had been a similar case but in a male patient published before: osteonecrosis and antiphospholipid in 2009 [11].

## Conclusions

Bone infarction remains an exceptional and unusual complication during primary APLS especially in young age complaining of mechanical joint pain. The revealing forms represent a real diagnostic challenge for clinicians. As rare as it is, this possible complication of primary APLS deserves to be known by health professionals and the diagnosis of primary APLS must be evoked in front of any bone infarction that is not proven by any other common causes, especially in any young woman.

Any young adult with even a single joint pain/limitation of movement especially those without suspected secondary causes should be investigated for APLS. Early treatment with anticoagulant would prevent serious disabling complications.

## Data Availability

Available.

## Abbreviations

NSAIDs: Non-steroidal anti-inflammatory drugs
ROM: Range of motion
EULAR: The European Alliance of Associations for Rheumatology
MRI: Magnetic resonance imaging
SLE: Systemic lupus erythematosus
APLS: Antiphospholipid syndrome
IBD: Inflammatory bowel disease
INR: The international normalized ratio
MFON: Multifocal osteonecrosis
